# Initiation of LPS-induced pulmonary dysfunction and its recovery occur independent of T cells

**DOI:** 10.1186/s12890-018-0741-2

**Published:** 2018-11-22

**Authors:** Eva Verjans, Stephanie Kanzler, Kim Ohl, Annette D. Rieg, Nadine Ruske, Angela Schippers, Norbert Wagner, Klaus Tenbrock, Stefan Uhlig, Christian Martin

**Affiliations:** 1 Department of Pediatrics, Medical Faculty, RWTH Aachen, Aachen, Germany; 2 Institute of Pharmacology and Toxicology, RWTH Aachen, Aachen, Germany; 3 Department of Anaesthesiology, Medical Faculty, RWTH Aachen, Aachen, Germany

**Keywords:** ARDS, Acute lung injury, T cell deficiency, Lung function, Lung mechanics, Lung inflammation

## Abstract

**Background:**

The acute respiratory distress syndrome (ARDS) is a serious disease in critically ill patients that is characterized by pulmonary dysfunctions, hypoxemia and significant mortality. Patients with immunodeficiency (e.g. SCID with T and B cell deficiency) are particularly susceptible to the development of severe ARDS. However, the role of T cells on pulmonary dysfunctions in immune-competent patients with ARDS is only incompletely understood.

**Methods:**

Wild-type (wt) and RAG2^−/−^ mice (lymphocyte deficient) received intratracheal instillations of LPS (4 mg/kg) or saline. On day 1, 4 and 10 lung mechanics and bronchial hyperresponsiveness towards acetylcholine were measured with the flexiVent ventilation set-up. The bronchoalveolar lavage fluid (BALF) was examined for leukocytes (FACS analysis) and pro-inflammatory cytokines (ELISA).

**Results:**

In wt mice, lung mechanics, body weight and body temperature deteriorated in the LPS-group during the early phase (up to d4); these alterations were accompanied by increased leukocyte numbers and inflammatory cytokine levels in the BALF. During the late phase (day 10), both lung mechanics and the cell/cytokine homeostasis recovered in LPS-treated wt mice. RAG2^−/−^ mice experienced changes in body weight, lung mechanics, BAL neutrophil numbers, BAL inflammatory cytokines levels that were comparable to wt mice.

**Conclusion:**

Following LPS instillation, lung mechanics deteriorate within the first 4 days and recover towards day 10. This response is not altered by the lack of T lymphocytes suggesting that T cells play only a minor role for the initiation, propagation or recovery of LPS-induced lung dysfunctions or function of T lymphocytes can be compensated by other immune cells, such as alveolar macrophages.

**Electronic supplementary material:**

The online version of this article (10.1186/s12890-018-0741-2) contains supplementary material, which is available to authorized users.

## Background

The acute respiratory distress syndrome (ARDS) is a life-threatening disease that is characterized by the rapid onset of severe respiratory failure with decreased pulmonary compliance, pulmonary inflammation and hypoxemia [1, 2]. It is frequently associated with sepsis, pneumonia and polytrauma [3] and the incidence of ARDS in the United States is 79 per 100,000 with a mortality of 40% [1]. Up to now, no pharmacological therapy is available that mitigates disease severity and/or mortality. The treatment remains largely supportive and the use of mechanical ventilation (MV) is mandatory [4–6].

ARDS commences with an inflammatory phase that is followed after 4–10 days by a fibroproliferative phase [7]. With respect to this paradigm, at least two important yet unsolved questions need to answered: (1) Because not all pulmonary inflammation leads to a measurable decline in physiological lung functions, the relationship between inflammation and lung functions deserves further study. (2) It remains unclear why some patients recover after the first phase while others enter the fibroproliferative phase [7]. The present paper addresses both questions by examining the role of T lymphocytes for LPS-induced lung dysfunction for 10 days.

During acute lung injury neutrophils are recruited well before lymphocytes [8, 9]. Based on these and other observations, it is thought that neutrophils are responsible for many of the pathophysiological alterations in the first phase, while T lymphocytes may be involved in the second-line defense and/or the recovery and resolution of inflammation. A recent study of BALF obtained from ARDS patients within 48 h of disease onset reported unchanged proportions of CD4 and CD8 lymphocytes and no increase in regulatory T cells (Treg), but found that T cells were activated (HLA-DR expression), proliferated (KI-67) and produced IL-17 [10]. A possible role of T cells in ARDS is further highlighted by patients with primary immunodeficiency (PID) with T cell dysregulation or absence. They show an enhanced susceptibility to pulmonary infections [11–13] that might be related to the overexpression of CREMα in the lymphocytes of these patients/mice [14].

A common way to study the role of lymphocytes in disease is the use of lymphocyte deficient mice. In LPS-induced lung injury at day 2 or later such studies have yielded conflicting results, i.e. fewer [8], similar [15] or higher numbers [16] of neutrophils in the BALF; similar inconsistencies were observed for lung injury scores, that were found to be either higher [16] or similar [17]. An important question in such models is the severity of the organ injury that can be assessed in a clinically relevant manner by lung function measurements. Up to date, such measurements had never been performed in lymphocyte-deficient mice.

Therefore, we first characterized lung mechanics and the associated inflammatory changes in LPS-induced lung injury for 10 days, i.e. for a time span long enough to observe the possible effects of lymphoyctes. Subsequently, we used RAG2^−/−^ mice to examine the impact of T cell deficiency on lung function parameters and disease activity during early and late phase of ARDS.

## Materials and methods

### Animals

Experiments were performed with 8 to 12 weeks old wild-type C57Bl/6 J mice (wt) and RAG2^−/−^ littermates, weighing 20 to 25 g. All mice were bred in our animal facility and kept under specific pathogen-free conditions. Room conditions were controlled for humidity (40–70%) and temperature (21–23 °C) with a 12-h light/dark cycle. Wild type and transgenic mice were age-matched for all experiments. The study was approved by regional governmental authorities and animal procedures were performed according to the German animal protection law and approved by regional governmental authorities (Landesamt für Natur, Umwelt und Verbraucherschutz Nordrhein-Westfalen, permission number: AZ 84-02.04.2016.A290).

### Experimental design and lung function measurements

Anesthetized mice (pentobarbital 70 mg/kg) were instilled intratracheally with an LPS (E.coli O111.B4, Sigma-Aldrich, Germany) aerosol (4 mg/kg) via a microsprayer (PennCentury, USA). Control animals received NaCl 0.9% and physiological parameters (body weight, behavior and temperature) were monitored in both groups during sleeping time of 20-30 min and the following 24-240 h. After 1, 4 or 10 days mice were tracheotomized with a 20G cannula and directly connected to the ventilator. All mice were initially anaesthetized with pentobarbital sodium (70 mg/kg) and fentanyl (0.1 mg/kg). Anaesthesia was maintained with pentobarbital sodium (20 mg/kg) after 30 min. All mice were mechanically ventilated with a tidal volume (Vt) of 10 ml/kg and a positive end-expiratory pressure (PEEP) of 2 cm H_2_O using the flexiVent (SCIREQ, Canada) ventilation setup. Body temperature was rectally controlled and adjusted between 36.5 and 37.5 °C during the whole ventilation period. Continuous data recording of heart rate and ECG was performed to monitor the function of the cardiovascular system.

Dynamic lung mechanics were measured by applying a sinusoidal standardized breath and analyzed with forced oscillation technique. We used a 1.2 s, 2.5 Hz single-frequency forced oscillation manoeuvre (SnapShot perturbation) and a 3 s, broadband low frequency forced oscillation manoeuvre containing 13 mutually prime frequencies between 1 and 20.5 Hz (Quick Prime perturbation). Total lung resistance (Rrs) and elastance (Ers) were calculated by the *flexiVent* software (flexiWare 7.0.1, SCIREQ, Canada) by fitting measured SnapShot values to the linear single compartment model using multiple linear regressions. Respiratory system input impedance was calculated from the QuickPrime data and tissue resistance (tissue damping, G) and tissue elastance (H) were assessed by iteratively fitting the constant-phase model to input impedance.

During the first 25 min of ventilation time baseline values were recorded using a standardized script with measurements every 30 s. Every 5 min short volume controlled recruitment maneuvers (deep inspirations over 3 s) were used to avoid atelectasis [18]. The data are presented as the maximum value obtained during these 25 min.

Following basal ventilation, airway hyperresponsiveness was provoked with nebulized acetylcholine (Ach). For each concentration lung function was measured 12 times (SnapShot and QuickPrime) during a period of 3 min.

After provocation of bronchial hyperresponsiveness, mice were sacrified by exsanguination via the carotid artery.

### Bronchoalveolar lavage and cytokine measurements

Following ventilation, lungs were removed. To obtain single lung cell suspensions, lungs were perfused with 5 ml sterile PBS through the right ventricle and the pulmonary artery at a constant hydrostatic pressure (15 cmH_2_O). The entire right lung was used for bronchoalveolar lavage fluid (BALF) by instilling 700 μL ice-cold PBS. Murine IL-6, TNF-α and KC were analyzed in supernatants of BALF samples with sandwich ELISAs according to manufacturer’s protocols (R&D Systems/eBioscience, Germany).

### FACS analysis

30 μL of BALF and 170 μL PBS/0.5% BSA were taken without staining to calculate absolute numbers of BALF cells with the BD LSR Fortessa analyzer (BD Bioscience, Germans). The remaining BALF was centrifuged for 10 min at 1250 × g and the pellet was resolved in 1 ml of PBS/0.5% BSA to wash the cells for a second time. After red blood cell lysis with lysis buffer, cells were stained with antibodies diluted in PBS/0.5% BSA for 20 min at 4 °C. For detection of T cells and the T cell subsets CD3-APC (eBioscience, Germany), CD4-PE (eBioscience, Germany), CD8-Pacific Blue and CD25-APC (eBioscience, Germany) were used. Neutrophil granulocytes were stained with Gr-1-FITC (Immuno Tools, Germany) and CD11b-Pacific Blue (eBioscience, San Diego). To identify alveolar macrophages, CD11c-APC-Cy7 (BD Bioscience, NJ, USA) and F4/80-PE (eBioscience, San Diego) were used. A minimum of 10,000 events were collected for evaluation.

### Statistical analysis

Time dependent data of body weight and temperature are shown as mean ± standard deviation (SD) and the area under the curve (AUC) was used for univariate analysis. All other data were presented as mean ± standard error (SEM). For all data, the Brown Forsythe test was used to check for equal variances and the BoxCox transformation was performed to achieve homoscedasticity if suitable. The ShapiroWilk test was used to verify normal distributions. For parametric data, differences between groups were tested using unpaired two-sided Student’s t-tests or ANOVA corrected by the Tukey post-test. Non-parametric data were analysed by Kruskall-Wallis test followed by Dunn’s post test. Graph generation and statistical analysis were performed by using Graph Pad Prism version 5.0 (GraphPad Software) or JMP 7.0.1 (SAS Institute). * *p* < 0.05, ** *p* < 0.01, *** *p* < 0.001.

## Results

### Wild-type animals

#### Body weight and rectal temperature

**Fig. 1:**
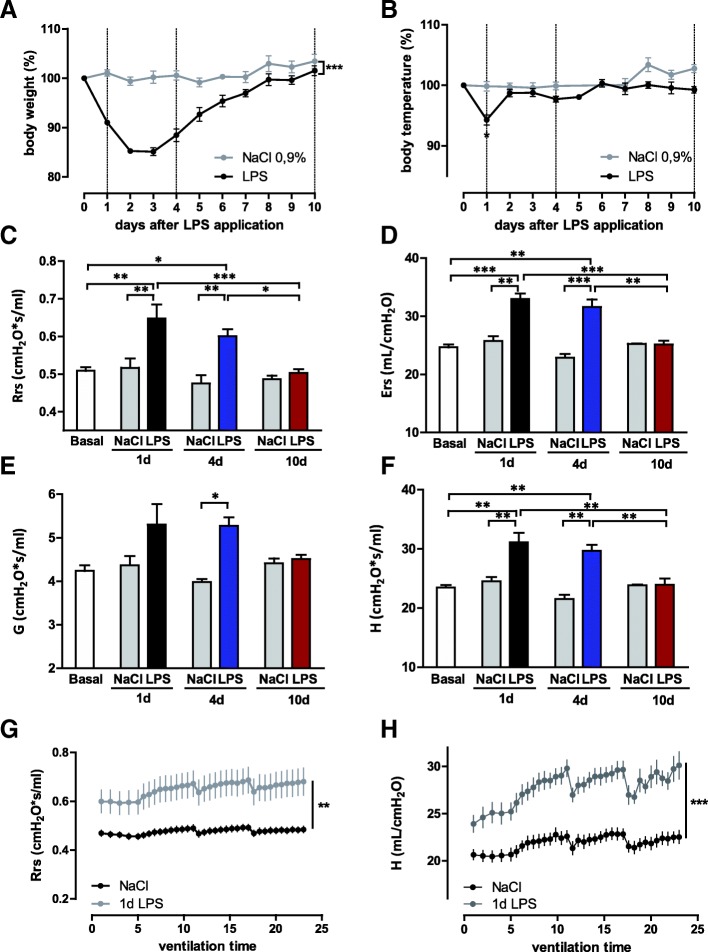
Characteristics of LPS-induced acute lung injury in C57BL6 mice. Mice were instilled with 50 μL of LPS (4 mg/kg) or saline (controls) on day 0 and were mechanically ventilated 1, 4 or 10 days after ARDS induction. Control mice were mechanical ventilated without prior i.t. instillation. ARDS disease activity was determined via body weight (**a**) and temperature (**b**) over the time (controls *n* = 4 and ARDS group *n* = 8 per time point, means ± SEM). **c**–**f** Lung function parameters resistance (Rrs), elastance (Ers), tissue damping (**g**) and tissue elastance (**h**) on day 1, 4 and 10 after ARDS-induction. (controls *n* = 4, ARDS group *n* = 6 per time point, means ± SEM). Lung function parameters over a ventilation time of 25 min, exemplary resistance (Rrs) and tissue elastance (H) of treated ARDS-animals and controls on day 1 are depicted in (**g**) and (**h**) (controls *n* = 12, summarized from all time points, ARDS group *n* = 8, means ± SEM). * *p* ≤ 0.05, ** *p* ≤ 0.01, *** *p* ≤ 0.001

Body weight and rectal temperature were followed for10 days. LPS-treated animals showed a continuous weight loss until day 3 and reached their initial body weight on day 9 to 10 (Fig. 1a). The NaCl-treated group developed normally (Fig. 1a). LPS treated mice showed a temperature drop on day 1, but not thereafter (Fig. 1b).

#### Lung mechanics

LPS-treated mice showed higher resistance (Rrs) and elastance (Ers) on day 1 and 4 after instillation and returned to baseline on day 10 (Fig. 1c–d). The resistance of the smaller airways (G) showed only slight differences between LPS-treated and control animals, whereas tissue elastance (H) was significantly increased on day 1 and 4 in the LPS-treated mice (Fig. 1e–f). Figure 1g–h exemplary presents lung mechanics over a ventilation time of 25 min on day 1 in comparison to control animals (Rrs and tissue elastance, H). Higher tissue elastance represents stronger stiffness of the smaller airways and therefore deteriorated lung function. Frequent increases in these graphs result from TLC manoeuvres every 5 min to avoid atelectasis [18]. As lung function parameters of saline-treated controls did not differ significantly at several time points after NaCl-instillation, these mice were summarized as control group in these time-dependent graphs for reason of clarity (Fig. 1c–d).

To study the occurrence of bronchial hyperresponsiveness (AHR), animals were provoked by 0.4 mg/kg Ach (Additional file 1: Figure S1A-D). Control animals responded only marginally to Ach, while ARDS mice showed a small increase in airway responsiveness towards methacholine (compare to Fig. 1c–f without acetylcholine).

#### BALF inflammatory mediators and cell counts

**Fig. 2:**
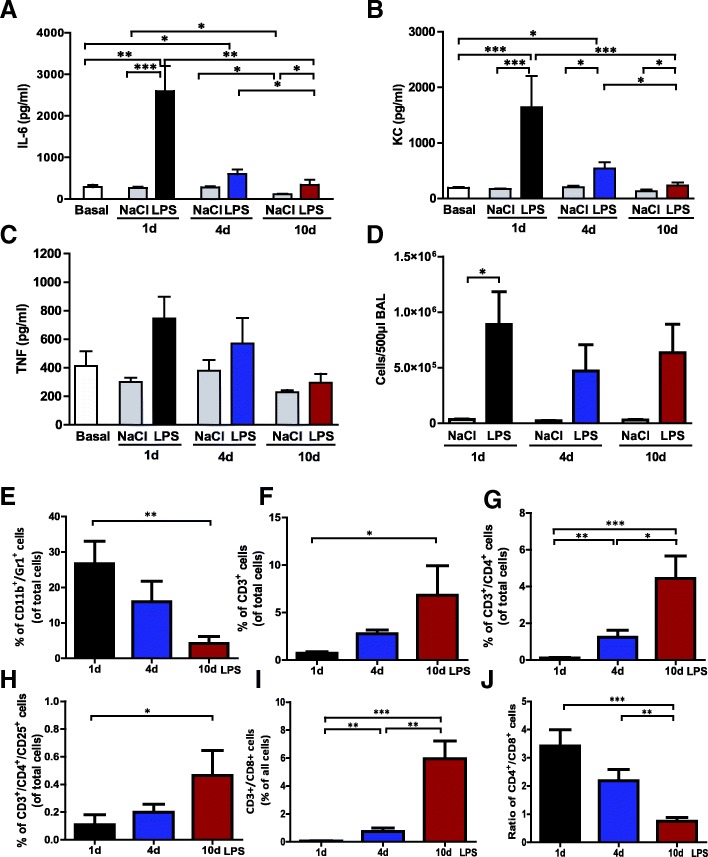
LPS induced inflammatory response in C57BL/6 mice. Proinflammatory cytokines IL-6 (**a**) and TNF-α (**c**) as well as the chemokine KC (**b**) were quantified in BALF supernatants on day 1, 4 and 10 after ARDS induction by ELISA. **d** Total cell numbers in BALF of LPS-treated animals and saline-instilled controls were calculated by FACS analysis. **e**–**j** Several cell subpopulations in ARDS mice were differentiated by specific antibodies, neutrophil granulocytes (**e**), T cell subpopulations (**f**–**i**) and CD4^+^/CD8^+^ ratio (F). Data are shown as means ± SEM. **a**–**j** Controls n = 4, ARDS groups *n* = 6. * *p* ≤ 0.05, ** *p* ≤ 0.01, *** *p* ≤ 0.001

Levels of IL-6 and KC peaked on day 1 after LPS-instillation and dropped during the following days (Fig. 2a–b). The amounts of TNF in BALF tended to be higher in ARDS-mice on all days, but its upregulation was not as high compared to the other cytokines (Fig. 2c).

As expected, levels of total cells in the BALF were much higher in LPS-treated animals than in control mice, but there were no significant differences in total cell counts (without lysis of erythrocytes) in different stages of disease (Fig. 2d). Therefore, we determined cell subpopulations in the LPS-treated groups. CD11b^+^ / GR-1^+^ positive cells were considered as neutropils; their levels were highest on day 1 after LPS-instillation and then continuously dropped until day 10 (Fig. 2e). In contrast, the amounts of T lymphocytes determined by total CD3^+^ cells as well as CD3^+^/CD4^+^, CD3^+^/CD4^+^/CD25^+^ and CD3^+^/CD8^+^ cells, increased over time. The ratio of CD4^+^/CD8^+^ cells clearly decreased from early to late stages of ARDS (Fig. 2f–j).

#### LPS-instillation in RAG^−/−^ mice

Our study demonstrated a strong increase of T cells during different phases of ARDS (Fig. 2f–i). To study the role of T cells in our model, we used RAG2^−/−^ mice that lack mature lymphocytes.

#### Body weight and rectal temperature

**Fig. 3:**
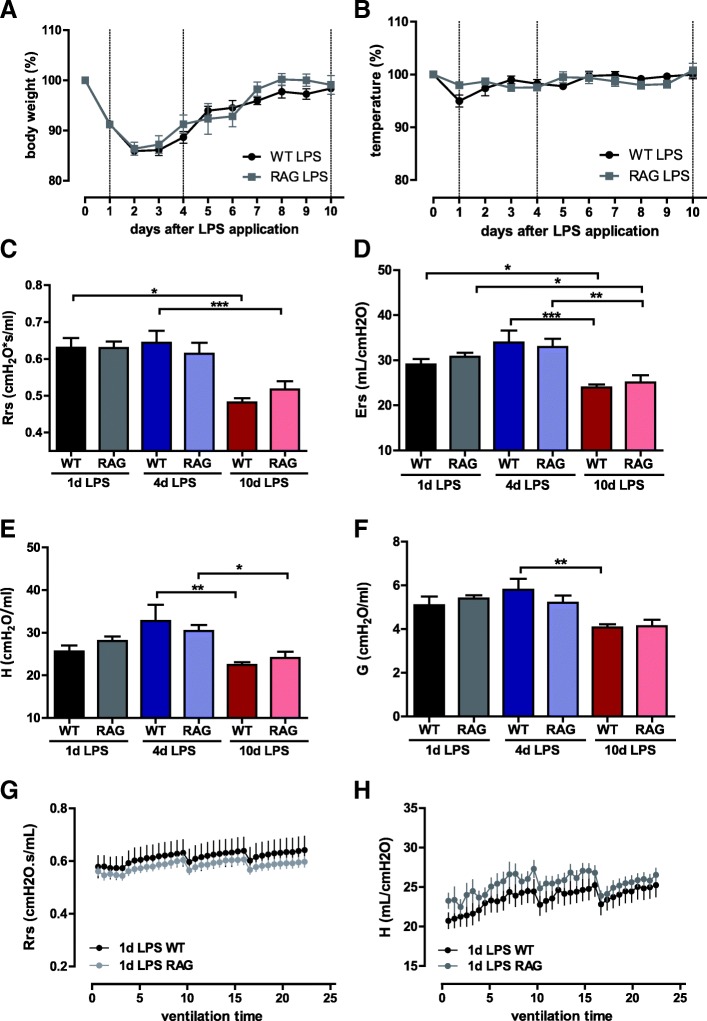
Characteristics of LPS-induced acute lung injury in RAG2^−/−^ and wildtype mice. ARDS was induced as described before in wildtype (WT) and RAG2^−/−^ (RAG) mice and animals were mechanically ventilated on day 1, 4 and 10 after ARDS induction. ARDS disease activity was determined via body weight and temperature over the time (**a**, **b**). **c**–**f** Lung function parameters resistance (Rrs), elastance (Ers), tissue damping (**g**) and tissue elastance (**h**) on day 1, 4 and 10 after ARDS-induction. Lung function parameters over a ventilation time of 25 min, exemplary resistance (Rrs) and tissue elastance (**h**) of treated ARDS-animals and controls on day 1 are depicted in (**g**) and (h). **a**–**h** (*n* = 8 in each group at each time point, means ± SEM). * *p* ≤ 0.05, ** *p* ≤ 0.01, *** *p* ≤ 0.001

Weight loss and the following weight gain as well as the temperature drop after LPS instillation were nearly the same in LPS-treated wt- and RAG2^−/−^ mice (Fig. 3a–b).

#### Lung mechanics

The lack of T lymphocytes in RAG2^−/−^ mice did not alter resistance and elastance of central and smaller airways on day 1, 4 or 10 after LPS instillation (basal ventilation) (Fig. 3c–h, g–h exemplary time courses on day 1). Further, there were no relevant differences in lung function parameters of RAG2^−/−^ and wt mice after provocation of bronchial hyperresponsiveness with acetylcholine (Additional file 2: Figure S2A-D).

#### BALF inflammatory mediators and cell counts

**Fig. 4:**
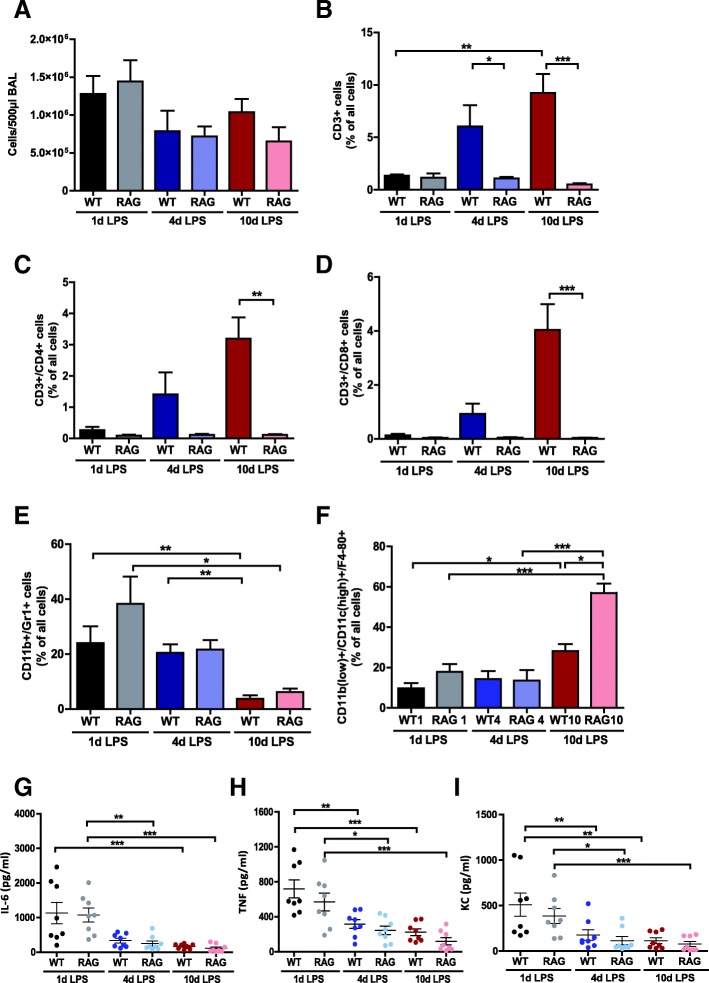
LPS induced inflammatory response in RAG2^−/−^ and wildtype mice. **a** Total cell numbers in BALF of LPS-treated RAG2^−/−^ animals and wildtype mice were calculated by FACS analysis. **b**–**f** Several cell subpopulations in ARDS mice were differentiated by specific antibodies. Proinflammatory cytokines IL-6 (**g**) and TNF-α (**h**) as well as the chemokine KC (**i**) were quantified in BALF supernatants on day 1, 4 and 10 after ARDS induction by ELISA. Data are shown as means ± SEM. **a**–**i** n = 8 per group, means ± SEM. * *p* ≤ 0.05, ** *p* ≤ 0.01, *** *p* ≤ 0.001

BALF Levels of IL-6, KC and TNF declined after day 1, both in wt and in RAG2^−/−^ mice. The total BALF cell counts were the same in wt and RAG2^−/−^ mice on day 1, 4 and 10 (Fig. 4a). Flow cytometric measurements demonstrated that all of the RAG2^−/−^ mice were lacking T lymphocytes: nearly no cells were counted in LPS-treated RAG2^−/−^ mice in the CD3^+^, CD3^+^/CD4^+^ and CD3^+^/CD8^+^ gates, whereas their wt littermates showed increasing numbers of different subpopulations of T lymphocytes over the time (Fig. 4b–d). Numbers of neutrophils (characterized by CD11b^+^/Gr1^+^) were not significantly different between wt and RAG2^−/−^ mice (Fig. 4e). Notably, the amount of CD11b^low +^/CD11c^high +^/F4–80^+^ cells (alveolar macrophages) was the same in both groups on day 1 and 4 but clearly differed on day 10 with 22% of total cells (wt) versus 57% (RAG2^−/−^) (Fig. 4f).

## Discussion

In a murine ARDS model lasting for 10 days we found that the major pathophysiological alterations – from inflammation to recovery – can occur independent of lymphocytes. In wt mice we observed the expected alterations in lung functions and inflammation during the early phase (up to day 4), and their recovery until day 10. Although pulmonary lymphocyte counts increased over the observational period of 10 days, mice lacking B and T lymphocytes (RAG2^−/−^), showed no relevant alterations in body weight, body temperature, lung functions, BALF cytokines or neutrophil counts compared to immune-competent wt littermates. Only the number of alveolar macrophages (CD11b^+^/CD11c^high +^/F4–80^+^) were higher in LPS-treated RAG2^−/−^ mice on day 10 and it may be speculated that these cells can compensate for the lack of lymphocytes during the recovery phase.

The instillation of LPS showed the expected time course with all changes being maximal between d1-d3, and recovery thereafter. The use of the constant phase model of lung mechanics allows to partition lung mechanics into a central airway component (R_N_) and a peripheral tissue component (G and H). The increase of G and H in LPS-treated mice therefore indicate increased stiffness and heterogeneity of the distal lung compartment including the small airways. We believe that lung function measurements are a highly useful readout in ARDS models, because they do not change in case of mild inflammation [18, 19] and because they provide an absolute measure that allows to compare the severity of lung injury across studies and with that of human ARDS. For instance, the loss of compliance in ARDS patients may typically be about 50% (e.g. 1.25 mL/cmH_2_O/kg BW [20] in anaesthetized healthy individuals vs 0.55 mL/cmH2O/kg BW in ARDS patients [21]), whereas in the present work it was roughly 30%, indicating that our lung injury was less severe than in ARDS patients. Based on these considerations we cannot exclude that lymphocytes play a role for the recovery or resolution in more severe ARDS.

The fact that all previous studies with lymphocyte deficient animals were lacking such measurements of compliance or other features of ARDS, makes it difficult to compare our studies to the previous ones.

According to the Berlin definition, there is no use of the term Acute Lung Injury (ALI) anymore. The committee felt that this term was used inappropriately in many contexts and hence was not helpful. In the Berlin definition, ARDS was classified as mild, moderate and severe according to the value of PaO_2_/FiO_2_ ratio. In our study, we do not reach ARDS according to this definition or we did not measure all necessary parameters according to PaO_2_/FiO_2_ ratio or chest radiation. However, our animals showed typical features of acute lung inflammation and a relevant tachypnoea. In human models, we would have assessed CPAP to our patients but this is not possible in our mouse model. Therefore, following the definition, we would classify our disease as milde ARDS or ARDS-typical acute lung inflammation.

ARDS is typically divided into at least two phases, where the acute phase is thought to be governed by the innate immune system, and later phases at least in part also by the adaptive immune system. As confirmed in the present work, the influx of T cells is usually low in the early phase, and rises until and during the recovery phase. According to our findings, however, this increase in lymphocyte has no bearing on the development or the recovery of the LPS-induced lung inflammation. Our findings in lymphocyte deficient RAG-2^−/−^ are in line with other studies showing unaltered lung injury in WT, nude and RAG-1^−/−^ mice [15, 17], but are in contrast to observations in RAG-1^−/−^ mice where inflammation was either less [8] or stronger [22, 23] and are also in conflict to the observation that pulmonary inflammation was increased in the absence of γδ T cells [24].

At present it is difficult to reconcile these contrasting findings for several reasons: (i) All these models used a similar model (LPS-administration via the airways), yet T-cell deficiency resulted in all possible outcomes, i.e. reduced, similar or increased lung injury; (ii) RAG1^−/−^ and RAG2^*−/−*^ are thought to possess nearly identical phenotypes [25]; (iii) T-cell dependent changes in TNF levels that have been proposed to explain the increased inflammation seen in γδ-knockout mice, were nearly the same in wt and RAG2^−/−^mice in the present study [24]. (iv) Treg cells that have been made responsible for the resolution of the LPS-induced inflammation, behaved as described [22, 23]: they increased towards d10 in wt mice and were lacking in the RAG2−/− mice. Thus, our findings seem to indicate that the recovery of inflammation is possible in the absence of γδ T cells and of regulatory T cells (which are not increased in human ARDS) [10]. Of course, these finding do not rule out the possibility that dysfunctional lymphocytes, such as those that overexpress CREMa, can exacerbate ARDS in both the acute and the recovery phase [14]. In general, the diversity of the published findings suggests that the role of lymphocytes during ARDS is highly context-dependent.

The only difference that we observed between wt- and RAG2^−/−^ mice was the number of alveolar macrophages (CD11b^+^/CD11c^high +^/F4–80^+^) that were increased in the lymphocyte-deficient mice on d10. It may be speculated that these cells could perhaps compensate T cell function [26], possibly through NOS expression [27]. Other cells that may organize the recovery and resolution of inflammation are M2 macrophages [28, 29] or even alveolar epithelial cells [30].

## Conclusion

In our model of LPS-induced lung injury, inflammation increased strongly while lung functions dropped by 30% within the first 3 days. Both inflammation and lung functions recovered until day 10 independent of the absence or presence of lymphocytes. These findings indicate that T cells play only a minor role for the initiation, propagation or recovery of LPS-induced lung dysfunctions. The many discrepant findings on the role of lymphocytes in ARDS suggest that their role is highly context-dependent and that further research in well defined and comparable models is required to improve our understanding of the role of the innate immune system in ARDS.

## Additional files

Additional file 1: **Figure S1.** Bronchial hyperresponsiveness in C57BL6 mice in LPS-induced acute lung injury**.** Mice were instilled with 50 μL of LPS (4 mg/kg) or saline (controls) on day 0 and were mechanically ventilated 1, 4 or 10 days after ARDS induction. Mice of basal group were mechanical ventilated without prior i.t. instillation. (A-D) Lung function parameters resistance (Rrs), elastance (Ers), tissue damping (G) and tissue elastance (H) on day 1, 4 and 10 after ARDS-induction following acetylcholine stimulation (controls *n* = 4, ARDS group *n* = 6 per time point, means ± SEM). * *p* ≤ 0.05, ** *p* ≤ 0.01, *** *p* ≤ 0.001. (PDF 311 kb)

Additional file 2: **Figure S2.** Bronchial hyperresponsiveness in RAG2^−/−^ and wildtype mice in LPS-induced acute lung injury**.** Mice were instilled with 50 μL of LPS (4 mg/kg) on day 0 and were mechanically ventilated 1, 4 or 10 days after ARDS induction. **(**A-D**)** Lung function parameters resistance (Rrs), elastance (Ers), tissue damping (G) and tissue elastance (H) on day 1, 4 and 10 after ARDS-induction following acetylcholine stimulation**)** (*n* = 8 in each group at each time point, means ± SEM). * *p* ≤ 0.05, ** *p* ≤ 0.01, *** *p* ≤ 0.001. (PDF 304 kb)

## Abbreviations

Ach: Acetylcholine
AHR: Airway hyperresponsiveness
ARDS: Acute respiratory distress syndrome
AUC: Area under the curve
BALF: Bronchoalveolar lavage fluid
CREM: CAMP-response element modulator
*E. coli*: *Eschericia coli*
ECG: Electrocardiography
ELISA: Enzyme-linked immunosorbent assay
FACS: Fluorescence-activated cell sorting
FoxP3: Forkhead box protein P3
IL-6: Interleukin 6
iNOS: Inducible nitric oxide synthase
KC: Keratinocyte chemoattractant
LPS: Lipopolysaccharide
MV: Mechanical ventilation
PBS: Phosphate buffered saline
PEEP: Positive end-expiratory pressure
PID: Primary immunodeficiency
RAG: Recombination-activating gene
SCID: Severe combined immunodeficiency
SD: Standard deviation
SEM: Standard error of the mean
TNF: Tumor necrosis factor
Vt: Tidal volume
WT: Wildtype
