# Supplementary figures and images for: Initiation of LPS-induced pulmonary dysfunction and its recovery occur independent of T cells
Source: BMC Pulm Med. 2018 Nov 22;18:174. doi: 10.1186/s12890-018-0741-2 (PMC6251177; doi:10.1186/s12890-018-0741-2)

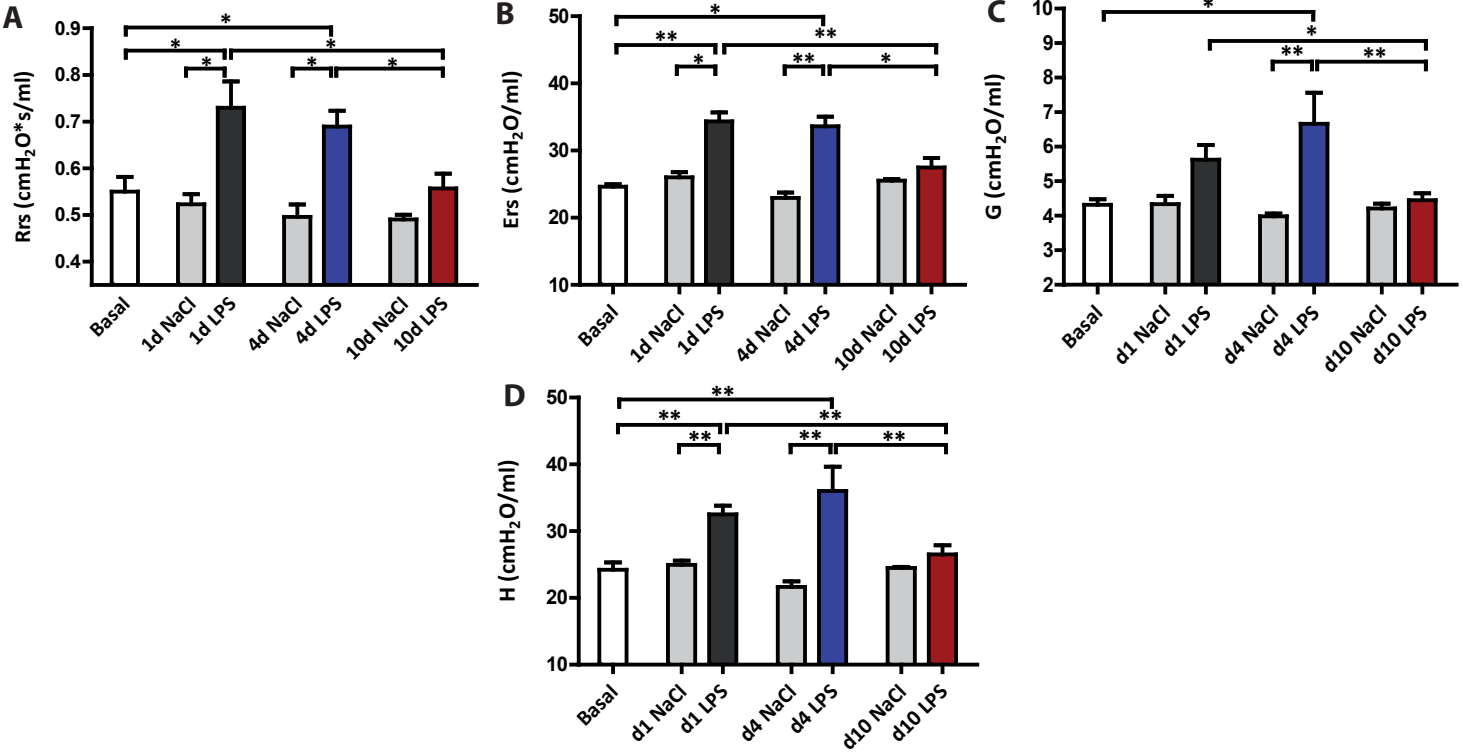

Supplement: Supplementary file 1 — Figure S1. Bronchial hyperresponsiveness in C57BL6 mice in LPS-induced acute lung injury. Mice were instilled with 50 μL of LPS (4 mg/kg) or saline (controls) on day 0 and were mechanically ventilated 1, 4 or 10 days after ARDS induction. Mice of basal group were mechanical ventilated without prior i.t. instillation. (A-D) Lung function parameters resistance (Rrs), elastance (Ers), tissue damping (G) and tissue elastance (H) on day 1, 4 and 10 after ARDS-induction following acetylcholine stimulation (controls n = 4, ARDS group n = 6 per time point, means ± SEM). * p ≤ 0.05, ** p ≤ 0.01, *** p ≤ 0.001. (PDF 311 kb) [file 12890_2018_741_MOESM1_ESM.pdf]

**A**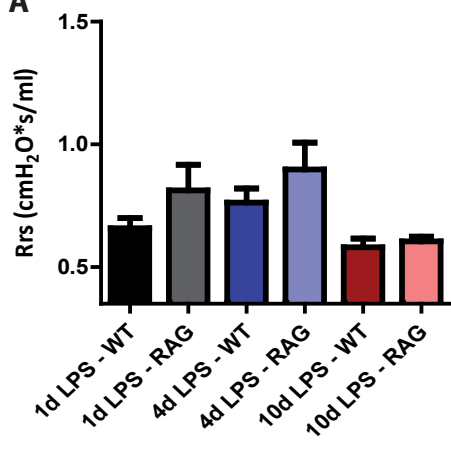**B**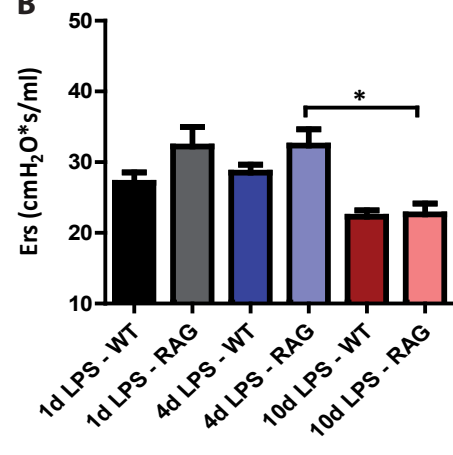**C**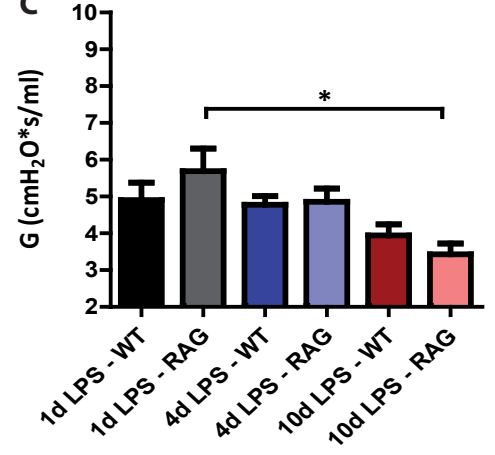**D**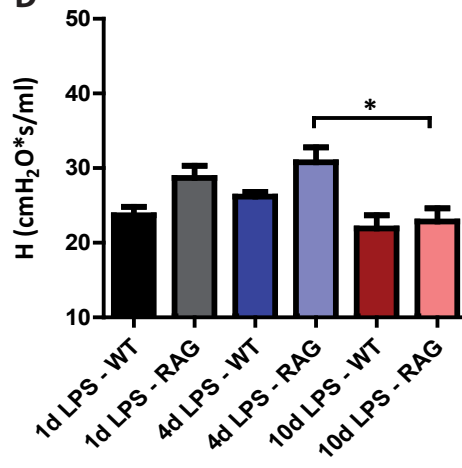

Supplement: Supplementary file 2 — Figure S2. Bronchial hyperresponsiveness in RAG2−/− and wildtype mice in LPS-induced acute lung injury. Mice were instilled with 50 μL of LPS (4 mg/kg) on day 0 and were mechanically ventilated 1, 4 or 10 days after ARDS induction. (A-D) Lung function parameters resistance (Rrs), elastance (Ers), tissue damping (G) and tissue elastance (H) on day 1, 4 and 10 after ARDS-induction following acetylcholine stimulation) (n = 8 in each group at each time point, means ± SEM). * p ≤ 0.05, ** p ≤ 0.01, *** p ≤ 0.001. (PDF 304 kb) [file 12890_2018_741_MOESM2_ESM.pdf]
